# Polyester 5–0 suture for porous implant placement after retinoblastoma enucleation: analysis of 120 sockets

**DOI:** 10.1186/s12886-023-02787-7

**Published:** 2023-01-23

**Authors:** Huijing Ye, Rongxin Chen, Wei Xiao, Xiufen Lian, Huasheng Yang

**Affiliations:** State Key Laboratory of Ophthalmology, Zhongshan Ophthalmic Center, Sun Yat-sen University, Guangdong Provincial Key Laboratory of Ophthalmology and Visual Science, Guangzhou, 510060 China

**Keywords:** Retinoblastoma, Sutures, Implant exposure

## Abstract

**Background:**

Techniques used to suture the rectus muscle to the implant can influence the implant-related complications which is still a major problem following retinoblastoma enucleation. The goals of this work were to report the efficacy among patients with retinoblastoma who underwent enucleation followed by porous implant placement with the rectus muscles sutured with 5–0 polyester suture.

**Methods:**

This was a retrospective study of consecutive patients with retinoblastoma who underwent primary enucleation and porous implant placement with the rectus muscles tagged and sutured to the implant with polyester 5–0 suture. All the patients were followed up for a minimum of 2 years. The main outcome measure was implant exposure. The secondary efficacy measures were other implant-related complications.

**Results:**

Between May 2016 and December 2018, a total of 120 patients (120 eyes) underwent primary enucleation and porous implant placement were included. Postoperatively, 10/120 (8.3%) eyes developed exposure or conjunctival granuloma. Exposure was the most common postoperative complication (7/10, 70.0%). There were no cases of implant extrusion, migration, or infection.

**Conclusions:**

Polyester 5–0 sutures are successful in patients with retinoblastoma who underwent enucleation followed by porous implant placement. Complications are minimal. Polyester 5–0 sutures were not associated with unacceptable complications in this pediatric population.

## Introduction

Despite progress in globe-saving treatment modalities for retinoblastoma, enucleation and insertion of a primary orbital implant, is still the preferred treatment of choice for advanced cases to provide acceptable cosmesis [1, 2]. Overall enucleation rate in intraocular retinoblastoma was 42.7% in one large series [3]. It’s worth noting that enucleation was still the first line treatment in 21.4% intraocular disease, mainly (40.1%) in advanced stages (groups D/E) [3, 4]. Meanwhile, secondary enucleation rate was reported as 27.2% following intravenous chemotherapy and/or focal treatments [3], 44.7% following intravenous and/or intra-arterial chemotherapy [5], and rare with naive bilateral eyes following intra-arterial chemotherapy [6]. The ideal implant should be easy to surgically implant and have low complication rates [7]. However, complications after enucleation such as socket contraction, migration, exposure or extrusion of the implant require revision surgery [8]. Exposure is the most common postoperative complication [9, 10]. Approximately 4.9–18.0% of retinoblastoma patients experience implant exposure, even with porous polyethylene implants, which has theoretical advantages and a favorable outcome over nonporous implants [8, 9, 11]. Thus, postoperative exposure is still a major problem following enucleation.

The techniques used to suture the rectus muscles can influence the exposure rate [12]. While absorbable sutures have a lower tensile strength and remain for only approximately two to 3 weeks in tissue, patients might experience implant extrusion or migration [9, 13]. Therefore, most authors prefer to use nonabsorbable sutures to suture surgical wounds [14]. Polyester sutures, as nonabsorbable sutures, have low tissue reactivity and excellent handling and tensile strength, and they retain suture strength in vivo for extended periods compared to other sutures [15, 16]. It has been reported that polyester sutures have been used to lock the four rectus muscles to the anterior surface of unwrapped hydroxyapatite orbital implants with a low exposure rate (2.0%) [17]. However, few comparisons of different sutures regarding the exposure of porous polyethylene implants have been conducted.

Hence, it seems worthwhile to evaluate the efficacy of polyester sutures for porous polyethylene implants in clinical use. In this study, we report the outcome of retinoblastoma in children who underwent enucleation and primary placement of porous polyethylene with polyester 5–0 suture to lock the rectus muscles to the anterior surface.

## Methods

### Study design

We retrospectively reviewed the records of all retinoblastoma patients who underwent enucleation and porous polyethylene implantation with polyester 5–0 suture between May 2016 and December 2018 and who had subsequently been followed-up for more than 2 years. Indications for perioperative chemotherapy were reduction of the intraocular tumor or high-risk pathologic features for orbital relapse and metastasis after enucleation. The exclusion criteria were secondary implantation, evisceration, death during follow-up, or follow-up of less than 2 years.

The patients’ demographic information, medical history, clinical and pathological findings, and complications were recorded. The main outcome was implant exposure. The secondary outcomes were other implant-related complications.

Approval was obtained in accordance with the Declaration of Helsinki and the Ethics Committee of Zhongshan Ophthalmic Center at Sun Yat-sen University.

### Surgical technique

The enucleations performed for the children for retinoblastoma were similar to previously described techniques using porous implants [9]. A 360° conjunctival periotomy was completed and Tenon’s layer was dissected away from the sclera. To separate Tenon’s capsule from the globe, the four quadrants between the rectus muscles were dissected bluntly, sparing as much of the conjunctiva and Tenon’s capsule as possible. All 4 rectus muscles were sequentially isolated with muscle hooks, secured with 5–0 polyester sutures (Polyester, Ethibond, Johnson and Johnson Health Care Systems, Piscataway, New Jersey, U.S.A.), and transected from the sclera. The superior and inferior oblique muscles were disinserted and allowed to retract into the orbit. Using traction on the medial rectus insertion site, the optic nerve was palpated and transected in the posterior orbit using long-tipped scissors. Hemostasis was obtained by compression of the socket with gauze.

A porous implantation (Medpor, Porex Technologies Co., Fairburn, GA) was placed as posteriorly as possible within the intraconal space using a sphere introducer. The implant size was chosen based on the largest metal sizer that could comfortably fit into the socket. Then each of the four rectus muscles was sutured directly to the sphere at the anterior surface of the implant (with a diameter less than 1 cm), with polyester 5–0 suture. Afterwards, we routinely closed both the posterior and anterior Tenon’s capsule over the sphere with interrupted 6–0 polyglactin sutures without excessive tension. Conjunctival closure was achieved using continuous or interrupted 6–0 polyglactin absorbable sutures.

### Statistical analysis

Statistical analysis was performed using SPSS for Windows (version 21.0, IBM Corp., Armonk, NY, USA). Comparisons of the effects of different factors on implant exposure were performed using the Student’s sample t test for continuous variables and chi-square test for categorical variables in a univariate risk factors analysis. The level of significance was set at *P* < 0.05.

## Results

Of the 125 consecutive patients who underwent primary enucleation and porous polyethylene implantation with polyester 5–0 suture during the study periods, five were excluded due to insufficient follow-up data died as a result of metastases. Therefore, a total of 120 patients who underwent primary enucleation and porous implantation were included in this analysis.

### Demographic and clinical characteristics

There were 66 (55.0%) male and 54 (45.0%) female patients. In total, 50 (41.6%) right eyes and 70 (58.3%) left eyes were treated. The median age at surgery was 25.5 months (range, 2–96 months). The median follow-up duration was 45.0 months (range, 25.0–58.0 months) after enucleation. The demographic and clinical characteristics are shown in Table 1. In all patients, the implants were placed unilaterally. The 20-mm sphere was the most common implant size in both groups.

**Table 1:** Demographics and clinical features of the study participants

Variable	Number of children (*N* = 120)
Age at surgery (months)	30.10 ± 18.63
Sex (male: female)	66:54
Laterality	
Unilateral	112 (93.33)
Bilateral	8 (6.67)
International Intraocular Retinoblastoma Classification	
Group D	21 (17.50%)
Group E	99 (82.50%)
Size of implant	
18 mm	20 (16.67%)
20 mm	97 (80.83%)
22 mm	3 (2.50%)
Preoperative chemotherapy	11 (9.17%)
Intravenous chemotherapy	8 (6.67%)
Periorbital chemotherapy	4 (3.33%)
Interventional chemotherapy	4 (3.33%)
Postoperative chemotherapy	47 (39.17%)
Intravenous chemotherapy	47 (39.17%)
Periorbital chemotherapy	0
Follow-up (months)	43.27 ± 9.29

### Postoperative complications

Complications of this procedure are listed in Table 2. Ten eyes (8.3%) experienced postoperative complications. Post-enucleation complications included implant exposure (*n* = 7, 5.8%), and conjunctival granuloma (*n* = 3, 2.5%). The 7 cases of exposure occurred in 3 females and 4 males, and one of them (14.3%) had symptoms of conjunctival hemorrhage. All implant exposures were repaired via implant revision and conjunctivoplasty and remained stable at median 31 months (range, 5–39 months) of follow-up. Only one exposure occurred in a child receiving preoperative chemotherapy, a known cause for poor wound healing. Three patients (2.5%) developed conjunctival granuloma with hemorrhage, which healed with granuloma resection. There were no cases of implant extrusion or allergic reaction to the polymer. Motility of the socket and fornices was excellent in all cases.

**Table 2:** Postoperative complications and outcomes

Complication	No. of Patients (%)	Interval from enucleation to complication (mos)	Age at enucleation (mos)	Treatment of complication
Implant exposure	7 (5.83)	20.86 ± 15.79	26.71 ± 11.50	Surgical repair
Conjunctival granuloma	3 (2.50)	43.00 ± 13.89	54.00 ± 26.89	Excision
Overall result of implant				
Success	120 (100)			
Failure	0 (0)			

Table 3 summarizes the clinical and treatment details of the patients and the analysis for the relative risk of complications. There were no significant associations between complications and sex, laterality, group of enucleated eye, age at enucleation, history of chemotherapy before or after enucleation, or implant size.

**Table 3:** Results of statistical analyses

Variable		All patients N (%)	Complications (*n* = 10)	No complications (*n* = 110)	*P*-value
Sex	Male	66 (55.00)	4 (40.00)	62 (56.36)	0.507
	Female	54 (45.00)	6 (60.00)	48 (43.64)	
Laterality	Unilateral	112 (93.33)	9 (90.00)	103 (93.64)	1.000
	Bilateral	8 (6.67)	1 (10.00)	7 (6.36)	
Group of enucleated eye	D	21 (17.50)	1 (10.00)	20 (18.18)	0.828
	E	99 (82.50)	9 (90.00)	90 (81.82)	
Age at enucleation (mean months ± SD)		30.10 ± 18.63	33.10 ± 20.40	29.83 ± 18.54	0.597
Pre-enucleation adjuvant chemotherapy	Systemic	8 (6.67)	1 (10.00)	7 (6.37)	1.000
	Subtenon’s carboplatin	4 (3.33)	0	4 (3.64)	1.000
Post-enucleation adjuvant chemotherapy	Systemic	47 (39.17)	4 (40.00)	43 (39.09)	1.000
Implant size (mean mm ± SD)		19.71 ± 0.83	19.40 ± 0.97	19.75 ± 0.81	0.210

## Discussion

The purpose of this study was to examine polyester 5–0 sutures for locking the rectus muscles to the porous implant and their influence on the rate of exposure and other complications in retinoblastoma patients. It has been confirmed that the techniques used to suture the rectus muscles can influence the exposure rate [17]. However, few comparisons of different sutures have been conducted for the subsequent exposure of porous polyethylene implants. In this paper, we present the results of our study on the application of polyester 5–0 sutures for orbital implants.

The incidence of unwrapped orbital implant exposure varies in the literature due to the use of different suture materials, including 5–0 polyester sutures (2.0–9.6%) [17], 5–0 or 6–0 Vicryl sutures (5–46%) [1, 9], and 5–0 catgut sutures (53.0%) [2]. In this case series, the authors found a reduced exposure rate (5.8%) in the 5–0 polyester suture cases, which is consistent with a previous report [17].

Absorbable sutures, including Vicryl, polyglactin, and catgut sutures, are also used to lock the four rectus muscles to the anterior surface of implants. However, there are some disadvantages regarding absorbable sutures. Polyglactin 910 sutures are associated with implant extrusion or migration with absorbable sutures [9, 13], partly because of the absorbable nature of the suture, which might lose its tensile strength, resulting in lower breaking points over time [18]. Patients have also experienced the development of stitch abscesses [15, 19]. Therefore, most authors prefer to use braided nonabsorbable sutures to close surgical wounds [14]. In contrast to absorbable sutures, polyester has an absolute higher breaking point [18]. As a result, none of our patients experienced implant extrusion or migration with nonabsorbable 5–0 polyester sutures in our study.

Moreover, patients with 5–0 polyester sutures in our study experienced a relatively long period from porous implant placement to exposure (20.86 ± 15.79 months). The time to exposure using absorbable sutures was as short as 136 days, ranging up to 12.5 months [1, 2]. The possible reasons for this include the fact that polyester sutures have higher knot and suture security and holding resistance, and nonabsorbable sutures are believed to be stronger [14]. In contrast, absorbable sutures, such as Vicryl, hold tensile strength only for approximately two to 3 weeks in tissue and are completely absorbed by hydrolysis within 56–70 days, typically with the following decay schedule: 75% at 2 weeks, 50% at 3 weeks, and 25% at 4 weeks [16].

Patients who were given pre-enucleation or post-enucleation chemotherapy have been proven to have an increased risk of complications [2, 8, 9]. However, this was not true in our study. We believe this might be because 5–0 polyester, which is a nonabsorbable suture, can strongly position the implant deep within the socket for a relatively long time compared to absorbable sutures. It has been proven that with the implant positioned deep within the socket, the tension on Tenon’s capsule and conjunctiva can be decreased [17].

It’s worth noting that several techniques may be used to try to minimize the complications after enucleation, including choosing an appropriately material type, appropriately sized orbital implant, posterior placement of the implant, muscle suturing technique, and meticulous closure of Tenon’s capsule and conjunctiva without tension [1, 17, 20]. Porous orbital implant, which was applied to our patients, has become the first choice for anophthalmic socket reconstruction in recent years with advantages over traditional alloplastic implants. Less implant migration and extrusion are found because of better fibrovascular ingrowth of microporous structure [21, 22]. The proper size of the implant, which is to allow tension-free closure of the anterior ocular tissue is also of great importance. Previous studies revealed a high incidence of exposure when larger implants are used [23].

The location of muscle attachment plays a role in occurrence of complications [24]. Compared with the technique of suturing muscles in our cases, myoconjunctival technique was also associated with minimal complications and provides acceptable cosmetic outcomes, including low implant exposure rate and good implant motility [25, 26]. However, the implant migration rate (9%) was still higher than in our cases, which could contribute to implant stability in the intraconal space [26]. Meanwhile, it was postulated that meticulous closing of Tenon’s and conjunctiva in separate layers generally gives a better mechanical closure, possibly less tension on the wound, and might reduce the risk of anterior surface breakdown [27]. In our cases, we closed both the posterior and anterior Tenon’s capsule over the sphere without excessive tension, guaranteeing a low complication rate.

Some limitations in this study need to be noted. This study was retrospective and follow-up duration was limited for some patients, which may have introduced some bias in the results. However, the median follow-up duration in our series was similar to or longer than the follow-up duration of most series in the currently available literature. The choice of suture used to lock the rectus muscles was based on the doctors’ personal experience with polyester or other materials. A randomized, prospective study comparing different sutures is lacking in the available literature but would offer additional information about the tolerance and complication rates of these types of sutures.

In conclusion, in the current study, we demonstrated that polyester 5–0 sutures are relatively safe in lowering the rate of implant exposure. This study may provide a novel and optimal suggestion for patients with retinoblastoma following enucleation that potentially results in few side effects.

## Data Availability

All the data used to support the findings of this study are included within the article and are available from the corresponding author upon reasonable request.
